# Stabilization of Intrinsically Disordered DKK2 Protein by Fusion to RNA-Binding Domain

**DOI:** 10.3390/ijms20112847

**Published:** 2019-06-11

**Authors:** Hye Min Lee, Soon Bin Kwon, Ahyun Son, Doo Hyun Kim, Kyun-Hwan Kim, Jonghyo Lim, Young-Guen Kwon, Jin Sun Kang, Byung Kyu Lee, Young Ho Byun, Baik L. Seong

**Affiliations:** 1 Department of Biotechnology, College of Life Sciences and Biotechnology, Yonsei University, Seoul 03722, Korea; wtingfm@naver.com (H.M.L.); yunbin829@gmail.com (S.B.K.); 50hyuny@naver.com (A.S.); flu1918h1n1@naver.com (Y.H.B.); 2 Vaccine Translational Research Center, Yonsei University, Seoul 03722, Korea; 3 Department of Pharmacology, and Center for Cancer Research and Diagnostic Medicine, IBST, School of Medicine, Konkuk University, Seoul 05030, Korea; eesli@naver.com (D.H.K.); khkim10@kku.ac.kr (K.-H.K.); 4 Department of Biochemistry, College of Life Science and Biotechnology, Yonsei University, Seoul 03722, Korea; jhyolim@naver.com (J.L.); ygkwon@yonsei.ac.kr (Y.-G.K.); 5 ProCell R&D Institute, ProCell Therapeutics, Inc., Ace-Twin Tower II, Guro3-dong, Guro-gu, Seoul 08381, Korea; jskang@procellrx.co.kr (J.S.K.); bklee@procellrx.co.kr (B.K.L.)

**Keywords:** DKK2, IDPs, RBPs, Wnt signaling, angiogenesis

## Abstract

Intrinsic disorders are a common feature of hub proteins in eukaryotic interactomes controlling the signaling pathways. The intrinsically disordered proteins (IDPs) are prone to misfolding, and maintaining their functional stability remains a major challenge in validating their therapeutic potentials. Considering that IDPs are highly enriched in RNA-binding proteins (RBPs), here we reasoned and confirmed that IDPs could be stabilized by fusion to RBPs. Dickkopf2 (DKK2), Wnt antagonist and a prototype IDP, was fused with lysyl-tRNA synthetase (LysRS), with or without the fragment crystallizable (Fc) domain of an immunoglobulin and expressed predominantly as a soluble form from a bacterial host. The functional competence was confirmed by in vitro Wnt signaling reporter and tube formation in human umbilical vein endothelial cells (HUVECs) and in vivo Matrigel plug assay. The removal of LysRS by site-specific protease cleavage prompted the insoluble aggregation, confirming that the linkage to RBP chaperones the functional competence of IDPs. While addressing to DKK2 as a key modulator for cancer and ischemic vascular diseases, our results suggest the use of RBPs as stabilizers of disordered proteinaceous materials for acquiring and maintaining the structural stability and functional competence, which would impact the druggability of a variety of IDPs from human proteome.

## 1. Introduction

A large number of human proteins are IDPs accounting for approximately 30–40% of the human proteome [1]. Intrinsic disorders are highly enriched in hub proteins in eukaryotic interactomes, allowing dynamic interaction with multiple partner proteins [2,3] in the context of cell signaling, transcriptional regulation, and pathogenic consequences if dysregulated [4,5]. Due to the structural instability without interacting partners, it is technically difficult to isolate or express IDPs or intrinsic disordered regions (IDRs) as a pure and structurally stabilized form, presenting a major challenge in target identification and validation as potential drug targets [6]. As such, most of successful drug targets are highly biased to well-structured proteins, amenable to functional validations.

**Figure 1:**
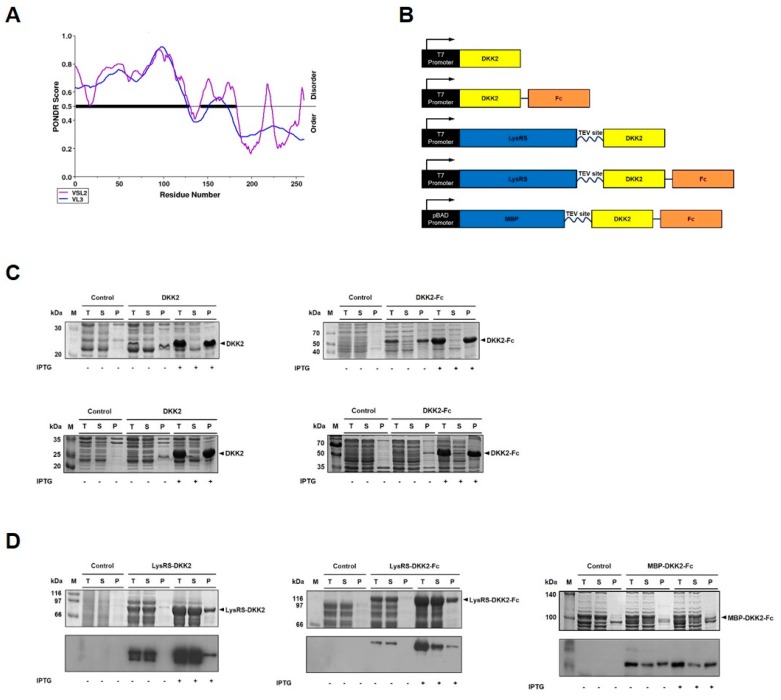
Expression of recombinant DKK2 protein in *E. coli*. (**A**) Disorder analysis of DKK2. Intrinsic disorder scores of DKK2 were predicted using PONDR^®^ software. The residues with scores above 0.5 were considered disordered. The thick black line comprising positions 1–129 and 142–182 highlights the disordered locations. (**B**) Schematic diagrams of expression constructs of DKK2, DKK2-Fc, LysRS-DKK2, LysRS-DKK2-Fc, and MBP-DKK2-Fc, respectively. (**C**) Expression of DKK2 or DKK2-Fc in *E. coli*. Proteins were expressed at 30 °C (upper panels) and 16 °C (lower panels). The solubility of proteins was analyzed by SDS-PAGE. T, S, and P indicate total lysate, soluble fraction, and pellet fraction after centrifugation, respectively. (**D**) Expression of DKK2 and DKK2-Fc with fusion to LysRS and DKK2-Fc with fusion to MBP in *E. coli*. The solubility of fusion proteins was analyzed by SDS-PAGE (upper panels) and western blotting (lower panels).

Wnt signaling is involved in regulating multiple biological processes for cell proliferation, survival, migration, and development of various tissues [7,8]. Wnt signaling is modulated by diverse receptors and their antagonists, including members of the Dickkopf (DKK) family of proteins, for angiogenesis, carcinogenesis, and osteoblastogenesis. Dysregulation of Wnt signaling has been implicated in multiple diseases, including cardiovascular and osteoarthritic disorders, and tumorigenesis [8,9,10,11,12]. Recent findings have shown that DKK2 plays similar functional roles to those of vascular endothelial growth factor (VEGF), the most well-characterized angiogenic factor [12,13]. DKK2 is induced during endothelial cell (EC) morphogenesis, promotes angiogenesis, and stimulates filopodial dynamics. Thus, the availability of recombinant DKK2 would facilitate the understanding of its regulation and dysregulation in ischemic vascular diseases [13], and angiogenesis-dependent tumor growth and metastasis [14,15,16,17,18,19] contributing to the treatment of cancer [20,21]. Despite functional analyses of this protein at the genetic, organ, and cellular level [9,10] since its discovery as a novel Wnt antagonist in 1999 [10], its characterization at the biochemical level is surprisingly absent, mainly due to structural disorder (Figure 1) and difficulties in establishing a system for acquiring the recombinant protein in vitro.

Compared with other expression systems, *Escherichia coli* (*E. coli*) is one of the most widely used prokaryotic organisms for the industrial production of proteins of therapeutic or commercial interest, with rapid growth at a high cell density on inexpensive carbon sources and simple scale-up ability [22,23]. Despite these distinctive advantages, most proteins from mammalian origins are expressed in *E. coli* predominantly as insoluble and non-functional aggregates (inclusion bodies), primarily due to lack of proper folding and post-modification systems. IDPs, due to their conformational repertoires, are able to interact with multiple partners and act as hubs in signal transduction pathways [3]. This dynamic nature of IDPs, although pivotal for providing a multi-functional role in vivo, presents difficulties in recombinant expression due to their lack of stabilized structure. Therefore, it is not surprising that recombinant DKK2 is highly unstable and prone to misfolding even in eukaryotic expression hosts, yeast or mammalian, rendering its functional validation extremely difficult (Kwon, Y.K.; Ha, I.H. personal communications). IDPs could be expressed by fusion to soluble partners, but the solubility never guarantees functional competence due to the presence of non-functional soluble aggregates [24]. 

We postulated that this problem could be circumvented by fusion of structurally unstable IDPs with stability enhancing partners. IDPs are highly enriched in RBPs [25,26], suggesting that RNA, as a ligand, may contribute to the stabilization if co-expressed with the target IDP. However, the identity of the RNA ligands is largely unknown. We, therefore, hypothesized that IDPs could be produced in a soluble and stabilized form by fusion to RBPs with well-established RNA ligands. As a proof-of-principle, we demonstrate that DKK2, in an Fc-fusion form aimed to increase its half-life [27], can be expressed in a soluble and biologically active conformation by fusion to RBPs that interacts with tRNA. Here, we chose to use LysRS as prototype RBP because of its pronounced ability to promote solubility of linked proteins in RNA-interaction dependent manner [28]. The recombinant protein exhibited activity as a Wnt-signaling antagonist, as well as an agonist for angiogenesis both in vitro and in vivo. Removal of LysRS prompted the formation of insoluble aggregates, suggesting a beneficial role of the RBP for maintaining solubility and activity of DKK2. The present work expedites not only the characterization of DKK2 as a novel therapeutic target in the Wnt-signaling-dependent signaling pathway, but also presents a robust platform for studying structurally ill-defined protein materials for biomedical applications.

## 2. Results

### 2.1. Expression and Purification of DKK2 Fusion Proteins

To identify IDRs in DKK2, we used the PONDR^®^ prediction algorithms VL3 and VSL2 [29,30]. We confirmed that DKK2 has highly disordered regions at amino acid positions 1–129 and 142–182 (Figure 1A). According to VSL2 analysis, 70.3% of DKK2 consists of IDRs. Since ~70% of mammalian proteins do not contain IDRs [31], DKK2 can be considered a highly disordered protein. Notably, the IDRs of DKK2 are distributed mainly in the N-terminus of the protein (Figure 1A). Considering the high degree of disorder, the propensity for structural destabilization is expected, especially without co-expression of binding partners. DKK2 and DKK2-Fc proteins were expressed in *E. coli* at 30 °C. The solubility of DKK2 and DKK2-Fc was analyzed by SDS-PAGE (Figure 1B,C). Low level ‘leaky’ expression was detected in the absence of isopropyl β-d-thiogalactopyranoside (IPTG) inducer, but the expression level was greatly increased in the presence of inducer (Figure 1C,D). We confirmed that both DKK2 and DKK2-Fc were expressed as insoluble aggregates. Low-temperature expression slightly increased the soluble yield, but insoluble aggregates still predominated (Figure 1C, lower panels). To circumvent this problem, DKK2 and DKK2-Fc were fused to the C-terminus of LysRS, a soluble folding enhancer, yielding LysRS-DKK2 and LysRS-DKK2-Fc protein, respectively (Figure 1B). The fusion proteins were expressed at 30 °C. The solubility of LysRS-DKK2 and LysRS-DKK2-Fc protein was determined by SDS-PAGE and western blot analysis (Figure 1D). We also compared maltose-binding protein (MBP) and LysRS fusion in the double domain fusion construct (DKK2-Fc) considering that the increase in the structural complexity would serve an acid test for structural stabilization. These results show that LysRS-DKK2 and LysRS-DKK2-Fc fusion proteins were expressed as soluble forms (80% and 85% of total protein, respectively). In contrast, fusion with MBP [32], a widely-used fusion partner for soluble expression, did rescue the solubility of DKK2, but only partially (~30% of total protein) (Figure 1D).

The expressed LysRS-DKK2-Fc protein was purified by Protein A affinity chromatography. Proteins in each fraction were analyzed by SDS-PAGE (Figure S1A), and eluted fractions were pooled and dialyzed against phosphate buffered saline (PBS). The purified protein was analyzed by SDS-PAGE and quantified (Figure S1B). The overall purification yield was ~2 mg from 1 L of *E. coli* culture.

### 2.2. Tobacco Etch Virus (TEV) Protease Cleavage of LysRS-DKK2-Fc

**Figure 2:**
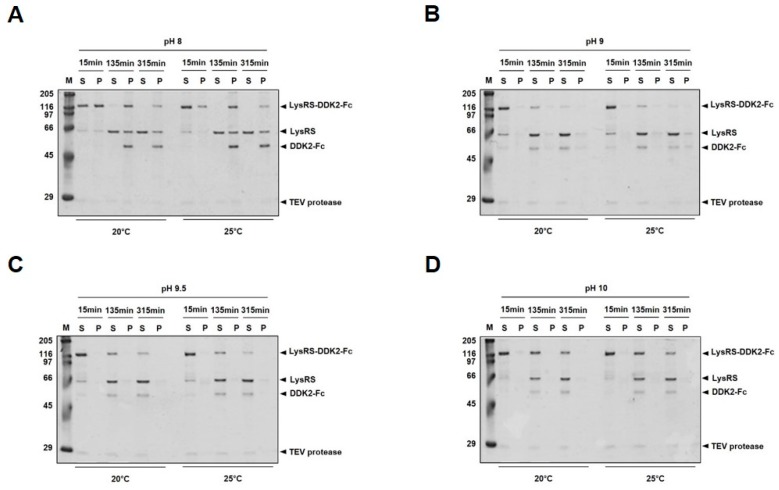
Cleavage of LysRS-DKK2-Fc by TEV protease. TEV protease cleavage of purified LysRS-DKK2-Fc in (**A**) pH 8.0, (**B**) pH 9.0, (**C**) pH 9.5 and (**D**) pH 10.0 buffer. The solubility of TEV-cleaved LysRS-DKK2-Fc protein was analyzed by SDS-PAGE.

**Figure 3:**
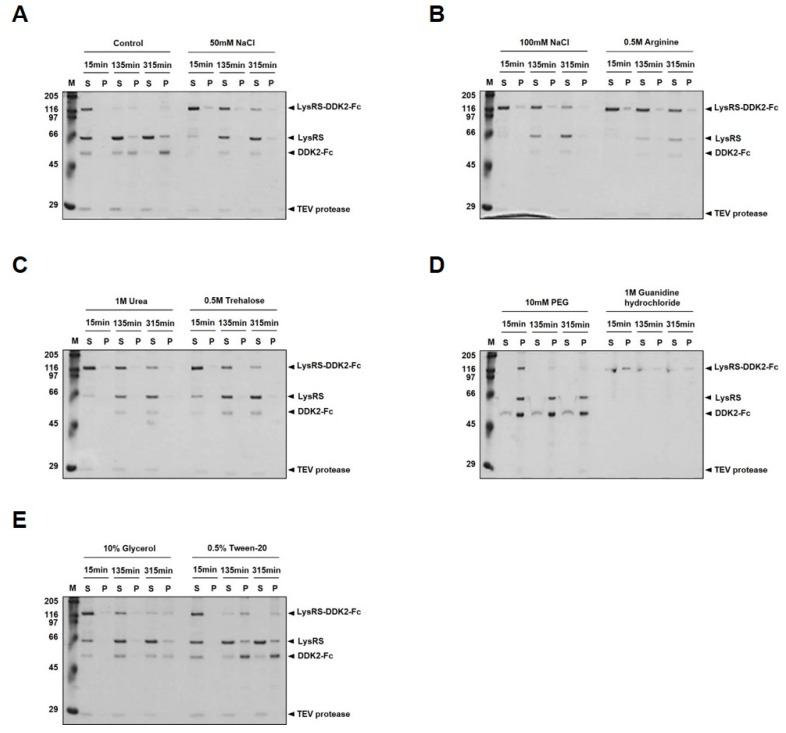
Effect of various excipients on TEV protease cleavage of LysRS-DKK2-Fc fusion protein. (**A**–**E**) The fusion protein was cleaved by TEV protease in the presence of various excipients known to influence protein solubility, including 50 or 100 mM NaCl, 0.5 M arginine, 1 M urea, 0.5 M trehalose, 10 mM polyethylene glycol (PEG), 1 M guanidine hydrochloride, 10% glycerol, or 0.5% Tween-20, in pH 9.0 buffer at 25 °C.

With technical difficulties in expressing and purifying DKK2/DKK2-Fc in sufficient quantities, we focused on LysRS-fusion form, for the rest of our experiments. We tested whether TEV protease efficiently cleaves the purified LysRS-DKK2-Fc fusion protein. As shown in Figure 1B, the fusion protein is composed of LysRS and DKK2-Fc, separated by a TEV protease cleavage site. To find the optimal condition for TEV protease cleavage of DKK2-Fc, we first applied TEV protease for up to 315 min in differing pH conditions at 20 and 25 °C. The efficiency of TEV cleavage was analyzed by SDS-PAGE (Figure 2A–D). The results show that 90–95% of LysRS-DKK2-Fc was cleaved by TEV protease at 20 and 25 °C after 315 min. We also found that the solubility of cleaved DKK2-Fc was maintained at high pH (pH > 9.5). However, the efficiency of TEV cleavage was low in pH 10.0 buffer (Figure 2D). We, therefore, determined the optimal cleavage condition to be pH 9.5 at 25 °C for up to 315 min. In addition, we tested various excipients known to be enhancers of protein solubility (Figure 3) [33,34,35,36,37,38,39,40]. We confirmed that trehalose improved the solubility of cleaved DKK2-Fc at ≥250 mM (Figure S2C). We further tested the stability of the fusion protein and TEV-cleaved protein in pH 9.5 buffer. Each protein was stored for up to five days at 4 and 25 °C. Consequently, we identified that the stability of the fusion proteins was well maintained when stored at 4 and 25 °C (Figure S3A,B), and cleaved DKK2-Fc was more stable at 4 °C compared to 25 °C (Figure S3C,D).

### 2.3. Recombinant DKK2-Fc Inhibits the Wnt Signaling Pathway

**Figure 4:**
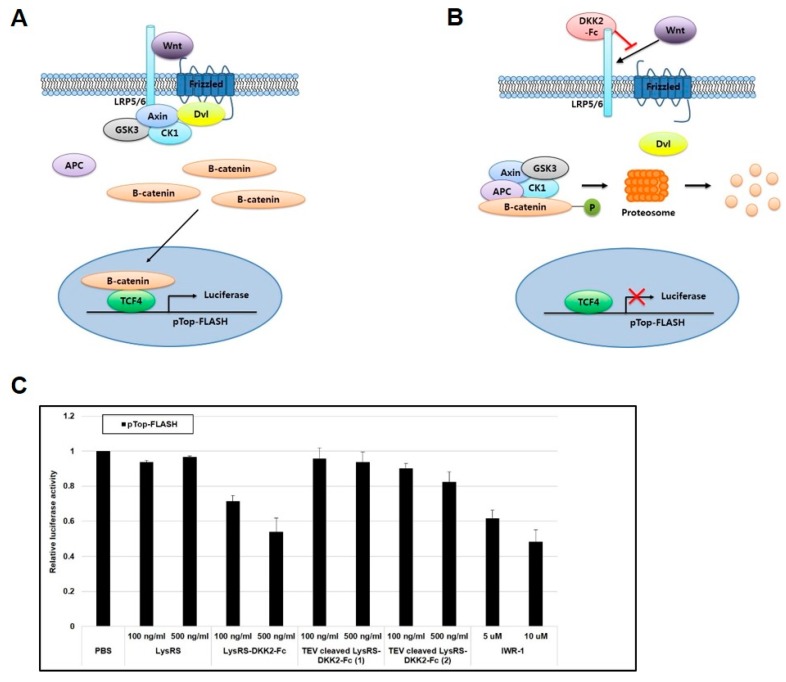

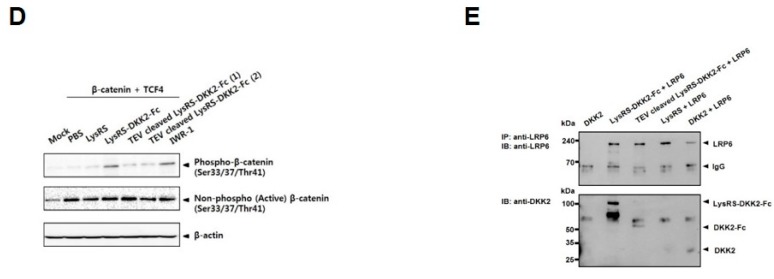
Inhibitory effect of recombinant DKK2-Fc on the Wnt signaling pathway. (**A**) Schematic diagram of the Wnt/β-catenin signaling pathway. (**B**) Schematic diagram of luciferase reporter gene transcription inhibition in the presence of recombinant DKK2-Fc. TOPflash reporter was co-transfected into HEK 293T cells with β-catenin and TCF4 plasmids. (**C**) TOPflash luciferase activity assay. HEK 293T cells were treated with PBS, recombinant protein (LysRS, LysRS-DKK2-Fc, TEV-cleaved LysRS-DKK2-Fc (1), TEV-cleaved LysRS-DKK2-Fc (2)), or IWR-1 as a positive control at two different concentrations. The bars indicate the relative TOPflash luciferase activity compared to that of PBS. The error bars indicate the mean ± SD of three experiments. (**D**) The effect of DKK2-Fc on phosphorylation of β-catenin in HEK 293T cells. HEK 293T cells were transfected with β-catenin and TCF4 plasmids, and were treated with PBS, recombinant protein (LysRS, LysRS-DKK2-Fc, TEV-cleaved LysRS-DKK2-Fc (1), TEV-cleaved LysRS-DKK2-Fc (2)), or IWR-1. Treated cells were collected, lysed, and subjected to western blotting using the indicated antibodies. (**E**) Co-immunoprecipitation of DKK2-Fc and LRP6. Each protein was immunoprecipitated with anti-LRP6 antibody and detected by anti-LRP6 or anti-DKK2 antibody.

DKK2 was originally identified as a Wnt antagonist that inhibits β-catenin signaling pathways. We used the TOPflash assay to examine if LysRS-DKK2-Fc or TEV-cleaved LysRS-DKK2-Fc inhibits Wnt signaling. TOPflash is a reporter plasmid containing T cell-specific factor 4 (TCF4) binding sites linked to the luciferase gene and is activated by Wnt signaling [20,41,42,43,44,45,46,47,48]. As shown in Figure 4A, in the presence of a Wnt ligand, Wnt binding to Frizzled (Fz) and low-density lipoprotein receptor-related protein (LRP) 5/6 receptor activates the Wnt pathway. Then, β-catenin moves to the nucleus where it associates with TCF4 to initiate luciferase reporter gene transcription. If DKK2 inhibits Wnt binding to LRP5/6, β-catenin would be phosphorylated, resulting in proteasomal degradation. In the absence of nuclear β-catenin, TCF4 proteins suppress luciferase reporter gene transcription (Figure 4B) [49,50,51]. 

Treatment of cells with LysRS-DKK2-Fc protein or IWR-1 inhibited TOPflash reporter activity (Figure 4C). IWR-1, a small molecule antagonist of the Wnt/β-catenin pathway, promotes β-catenin degradation by promoting the stability of Axin [41,46]. Thus, it was used as a positive control. The results show that 500 ng/mL (4.2 nM) LysRS-DKK2-Fc and 10 µM IWR-1 inhibited 47% and 52% of TOPflash reporter activity, respectively. In contrast, PBS and LysRS had little effect, if any, on TOPflash reporter activity. TEV-cleaved LysRS-DKK2-Fc (1) refers to LysRS-DKK2-Fc that was treated with TEV protease at 25 °C for 315 min and added to cells. TEV-cleaved LysRS-DKK2-Fc (2) refers to LysRS-DKK2-Fc mixed with TEV protease and immediately added to cells. TEV-cleaved LysRS-DKK2-Fc (1) did not inhibit TOPflash reporter activity, while 500 ng/mL of TEV-cleaved LysRS-DKK2-Fc (2) inhibited 18% of TOPflash reporter activity. 

We further tested the effect of recombinant DKK2-Fc on Wnt/β-catenin signaling by monitoring the levels of phosphorylated β-catenin by western blotting (Figure 4D). Binding of β-catenin with the Axin-based multiprotein complex leads to the phosphorylation of β-catenin [12,51,52,53,54]. Phosphorylated β-catenin was clearly detected in both recombinant LysRS-DKK2-Fc- and IWR-1-treated human embryonic kidney (HEK) 293T cells. Endogenous levels of non-phosphorylated β-catenin were detected by non-phospho (active) β-catenin (Ser33/Ser37/Thr41) antibody. These results indicate that recombinant DKK2-Fc had functional activity as a Wnt antagonist in Wnt/β-catenin signaling. 

To identify if the interaction between recombinant DKK2-Fc and LRP6 is direct, we performed co-immunoprecipitation of DKK2 with LRP6 (Figure 4E). Recombinant LysRS-DKK2-Fc, TEV-cleaved LysRS-DKK2-Fc, and DKK2 were precipitated with LRP6. Moreover, DKK2 was not detected in the absence of LRP6. Thus, we confirmed that the antagonist function of recombinant DKK2-Fc is LRP6-mediated.

### 2.4. Recombinant DKK2-Fc Induces Tube Formation in an In Vitro Model of Angiogenesis

**Figure 5:**
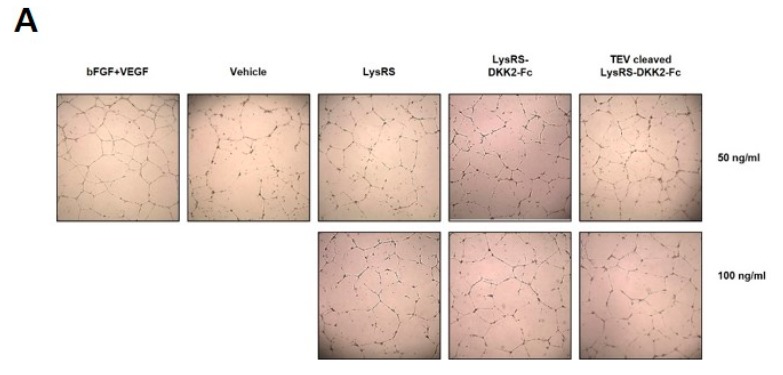

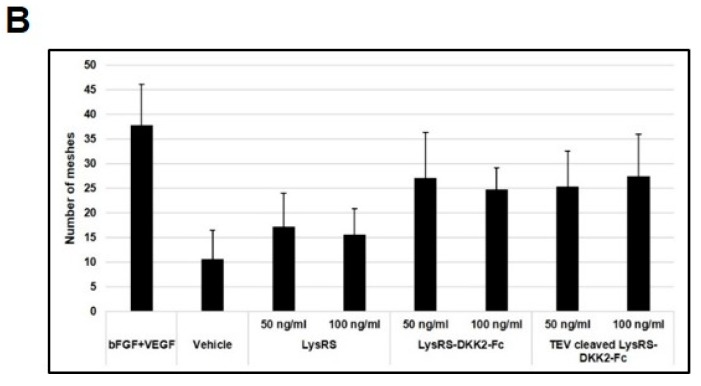
Effect of recombinant DKK2-Fc on the formation of EC tube-like structures. (**A**) Tube formation assay using HUVECs. HUVECs were incubated on Matrigel with 50 or 100 ng/mL recombinant proteins (LysRS, LysRS-DKK2-Fc, or TEV-cleaved LysRS-DKK2-Fc), or with a positive control consisting of a mixture of 20 nM bFGF (75 ng/mL) and 10 nM VEGF (270 ng/mL), for 16 h, and observed microscopically (4×). (**B**) Visual counts of the number of branch points were performed independently by three individuals.

DKK2 is known to induce EC morphogenesis and stimulate angiogenic sprouting [13]. We tested the biological function of LysRS-DKK2-Fc and TEV-cleaved LysRS-DKK2-Fc in vitro with a tube formation assay in HUVECs. When Matrigel-containing media was added to a monolayer of HUVECs, the attached ECs formed branched tubes. HUVEC monolayers treated with 50 or 100 ng/mL recombinant LysRS-DKK2-Fc and TEV-cleaved LysRS-DKK2-Fc for 16 h resulted in significant enhancement of EC tube-like structures (Figure 5A,B). Recombinant DKK2-Fc was shown to have 65–75% activity compared with basic fibroblast growth factor (bFGF) and VEGF, which are potent angiogenic factors. Negative controls vehicle and LysRS had no detectable angiogenic activity (Figure 5A,B). These results indicate that DKK2-Fc had a biologically active conformation and significant functional activity during angiogenesis in vitro.

### 2.5. Effect of Recombinant DKK2-Fc on Angiogenesis In Vivo

**Figure 6:**
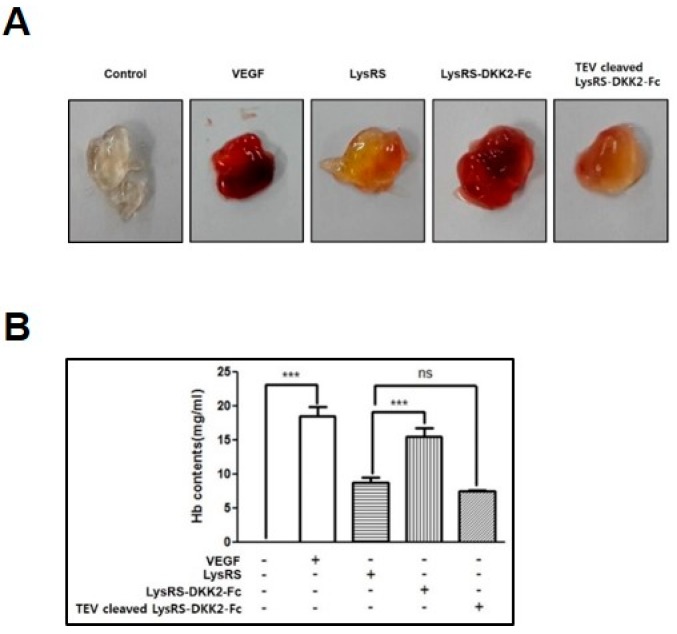
Recombinant DKK2-Fc induces angiogenesis in Matrigel plugs. (**A**) C57BL/6 mice were injected with 0.6 mL Matrigel containing VEGF, LysRS, LysRS-DKK2-Fc, or TEV-cleaved LysRS-DKK2-Fc. After three days, Matrigel plugs were excised from mice. (**B**) Quantification of Hb in Matrigel plugs. Error bars are the mean ± SD of three experiments in duplicate. *** *p* < 0.001; ns, not significant.

To assess the effect of LysRS-DKK2-Fc and TEV-cleaved LysRS-DKK2-Fc on angiogenesis in vivo, we utilized the Matrigel implant assay in mice [13]. Mice were implanted subcutaneously with 14 nM LysRS-DKK2-Fc- or TEV-cleaved LysRS-DKK2-Fc-treated Matrigel plugs. The positive control was 14 nM VEGF, and LysRS and PBS served as negative controls. After three days, the mice were sacrificed, and the plugs were recovered. As shown in Figure 6A, control plugs that were treated with PBS appeared transparent, indicating the absence of angiogenesis, whereas VEGF- or LysRS-DKK2-Fc-containing plugs exhibited a red color, indicating that LysRS-DKK2-Fc induced vessel development in the plugs. TEV-cleaved LysRS-DKK2-Fc- and LysRS-containing plugs appeared to be light red and/or yellowish in color, indicating that TEV-cleaved LysRS-DKK2-Fc and LysRS induced low levels of neovascularization compared with the positive control. 

Quantification of the functional vasculature by determining the Hemoglobin (Hb) content of the Matrigel plugs indicates that recombinant LysRS-DKK2-Fc significantly promoted angiogenesis compared with the controls, while TEV-cleaved LysRS-DKK2-Fc had low angiogenic activity in vivo (Figure 6B).

## 3. Discussion

A large portion of human proteome are represented by IDPs and has been implicated in numerous diseases, making them potential targets for therapeutic intervention [3,55]. However, most of the successful drug targets are represented predominantly by well-structured proteins, only to belie technical hurdles in functional validation of IDPs as viable drug targets. Intrinsically dynamic nature of IDPs or IDRs is requisite for allowing multiple protein–protein, protein–DNA, and protein–RNA interactions [3,5,56]. As such, the isolation and characterization of individual, purified IDPs as functionally stabilized form remains a challenge. Guided by in silico prediction that IDPs are highly enriched in RBPs and DNA-binding proteins (DBPs) [25,26,57,58], here we show that, DKK2, with a high propensity to disorderedness (Figure 1A), could be stabilized by fusion to RBP, and become amenable to biochemical validation in vivo and in vitro. Here, we chose to use LysRS as prototype RBP because of its excellent ability to increase the solubility of linked proteins, which operates in an RNA-interaction dependent manner. First, tRNA synthetases including LysRS are able to interact with various tRNAs in the cytoplasm at low affinity [59]. Second, when linked to aggregation-prone proteins, LysRS potently enhances the solubility of a variety of proteins of mammalian origin [28]. Third, LysRS itself folds in tRNA-interaction dependent manner in vitro suggesting that tRNA functions as a chaperone for its interacting tRNA synthetase [28].

The biochemical characterization of DKK2 would facilitate detailed analyses of its role in Wnt signaling and dysregulation into ischemic vascular diseases, and cancer [13,20,21]. However, due to the lack of biochemical information and efficient recombinant expression platforms, little progress has been made so far on utilizing the unique functions of this protein. The technical difficulties are probably reflected in the tendency of DKK2 to aggregate in the absence of stabilizing partners. Of note, DKK2 has a high isoelectric point (pI ~ 9.37) and is highly disordered (overall score >70% by PONDR^®^), with IDRs present in the majority (N-proximal 1–180 residues) of whole protein (Figure 1A). While the structure of the relatively small C-terminal region (amino acids 172–259) of mouse DKK2 was investigated by NMR spectroscopy [60], no information on the overall structure is available. The paucity of structural information to date reflects the technical difficulties encountered with the structural disorder encompassing the majority of DKK2, supporting our prediction in Figure 1A.

Consistent with this, our results show that DKK2 is prone to misfolding into inclusion bodies, and refractory to soluble expression in *E. coli* (Figure 1C). This problem was effectively circumvented by creating a LysRS-DKK2 fusion protein. Notably, the fusion protein was expressed in *E. coli* predominantly as a soluble form (>80% of solubility), either with or without Fc fusion (LysRS-DKK2-Fc and LysRS-DKK2, respectively) (Figure 1D). Fusion to MBP [32], a widely-used partner for soluble expression, was only partially effective in improving the solubility of DKK2 (~30% of solubility) (Figure 1D). The acquisition of DKK2 as a soluble form allowed for its detailed characterization at the biochemical level. We found that stable maintenance of protein solubility was crucially dependent on buffer conditions. The solubility of DKK2-Fc, after TEV cleavage of LysRS-DKK2-Fc, was maintained at higher pH (>9.5), but significantly reduced at lower pH (<9.0) (Figure 2). Our initial data on the physicochemical properties warranted further testing of various excipients to determine the optimal formulation conditions with respect to the stability and pharmacokinetic profile of DKK2. Among the excipients tested, trehalose (0.5 M) was only partially effective at maintaining solubility (Figure 3). 

Recombinant LysRS-DKK2-Fc was biologically active in inhibiting the Wnt signaling pathway as determined by TOPflash assay (Figure 4C). DKK2 is known to interfere with the Wnt pathway by binding to the Wnt co-receptors LRP5/6, leading to the disassembly of the Wnt/Fz/LRP complex, and the phosphorylation of β-catenin, resulting in proteasomal-dependent degradation [49,61,62,63]. Consistently, the treatment of LysRS-DKK2-Fc resulted in the phosphorylation of β-catenin (Figure 4D), and consequently, the downregulation of the luciferase reporter (Figure 4C). Furthermore, binding of LRP6 with LysRS-DKK2-Fc was confirmed (Figure 4E). Removal of the LysRS moiety by TEV protease resulted in a rapid loss of solubility (Figure 2), suggesting that LysRS keeps DKK2 in a soluble and biologically competent form. The low solubility may reflect the low activity of cleaved DKK2 in the TOPflash assay and in vivo Matrigel implant experiment (Figure 4C,D and Figure 6). LysRS-DKK2-Fc was cleaved by TEV. However, it still showed biological function in some experiments (Figure 4E and Figure 5), indicating that the stability of this protein is more sensitive to experimental conditions compared to LysRS-DKK2-Fc. Of note, the activity of 4.2 nM LysRS-DKK2-Fc was similar to that of 10 µM IWR-1, a well-known Wnt antagonist [41,46]. This result indicates that LysRS-DKK2-Fc has strong potential as a novel anticancer drug target. 

LysRS-DKK2-Fc was also shown to be active in the formation of EC tube-like structures in induced angiogenesis, based on Matrigel assay both in vitro and in vivo (Figure 5 and Figure 6, respectively). Previous studies have shown that angiogenic sprouting is induced by DKK2, which promotes Rho GTPase Cdc42 activation in vascular morphogenesis and ECs [13], and LRP6, as a DKK2 receptor, mediates Cdc42 activation and angiogenesis. The binding activity of LRP6 with LysRS-DKK2-Fc in vitro (Figure 4E) is therefore consistent with EC tube formation in vitro and angiogenesis in vivo (Figure 5 and Figure 6, respectively). It should be noted that the angiogenic activity of LysRS-DKK2-Fc was comparable to that of the VEGF control, as determined both visually and by quantitative Hb content (Figure 6A,B, respectively). The fusion to LysRS was found to be essential for vascular formation in vivo, further confirming similar observations of Wnt-signaling inhibitory activity (Figure 4). Overall, our results suggest that LysRS provides functional competence to the physically linked DKK2 as reflected by the solubility and more importantly, the biological activity of DKK2, which otherwise renders unstable and misfolds into an inactive form. 

Considering interdependence for mutual structural stabilization of IDPs among binding partners [2,3], expression and purification of an IDP of interest alone in a stabilized form present a major challenge for their structural and biochemical characterization. The present report circumvented the problem by linking the protein material with an RBP, which is consistent with the enrichment of IDPs in naturally occurring RBPs [25]. Moreover, there is accumulating evidence that RNA-protein complexes form higher-dimensional structures or lattices, providing territories for phase transitions [64]. This provides for the possibility for IDPs to be produced in functionally stabilized forms by co-expression with their RNA ligands. In most cases, however, the identity of the RNA ligand is not known, rendering RNA co-expression difficult. An alternative is, as presented in the present report, to fuse the IDP of interest with known RBPs or RNA-binding domains (RBDs), such as LysRS that interacts with tRNA. 

We note that the present results are limited and, therefore, discretion should be given to the extent of interpretation. First, a variety of IDPs and RBPs should be tested before generalization. However, our limited experience with other IDPs (PONDR^®^ score > 0.5, 45~55%), for instance, hepatitis B virus X protein (HBx) of viral origin [65], ribosomal protein S3a (RPS3a) of mammalian origin [66], which are themselves refractory to soluble expression, are all excellently solubilized by linking to LysRS (unpublished results). PB1-F2 with distinctive internal IDR region, and a novel virulence factor for 1918 Spanish influenza pandemic was successfully purified as LysRS fusion, and its antiviral function validated [67]. Second, the mechanistic aspect for chaperoning the functional competence should merit further investigation. There are at least two different possibilities. First, target IDP may interact with IDR region of RBPs for structural stabilization. Of note, LysRS contains an IDR component connecting structurally stable N-terminal and C-terminal domains [68]. Second, structural stabilization is mediated by an RNA component that interacts with RBPs. It should be noted that RNAs are able to make multiple contacts with hydrophobic side chains of polypeptide, as molecular signatures for ‘hydrophobic shielding’ of the folding intermediates, a hallmark function of molecular chaperones [69,70]. It remains to be further elucidated if tRNA-dependent folding of LysRS observed in vitro [28] also operates in LysRS-fusion protein in vivo as well. Finally, a cautionary note should be given to the relationship between solubility and biological function of IDPs. Although various fusion partners (MBP, N utilization substance A (NusA), glutathione-*S*-transferase (GST), small ubiquitin-related modifier (SUMO), and RBD fusion as the present case) can be mobilized to enhance the solubility of target proteins, the solubility never guarantees the function. Discretionary analyses are required to ascertain functional form from non-functional soluble aggregates [24]. 

Growing evidence shows that RNAs can function as molecular chaperones for the folding of nascent polypeptides and maintain their in-cell solubility and/or stability [28,71], surprisingly out-performing protein-based chaperones [28,70,72,73]. As such, pre-existing molecular chaperones, such as GroEL/ES and DnaK, have been applied with only limited success [69,74,75], and no study of considerable size showing the utility of molecular chaperones for the preparation of human proteins, including IDPs [76]. Limited it may be, the present report suggests RBP fusion as an initial screen for the preparation of structurally unstable protein materials. The optimal combinations of IDPs and RBDs could be tailored for increasing the solubility, stability, and the functional competence of the aggregation-prone disordered proteins.

## 4. Materials and Methods

### 4.1. In Silico Prediction of DKK2 Disorder Regions

DKK2 disorder regions were analyzed using the online PONDR^®^ software [29,30]. A disorder score for each amino acid was assigned by the software. Residues with score values above 0.5 were considered to be disordered. 

### 4.2. Construction of Protein Expression Plasmids

The expression plasmids pGE-LysRS1-DKK2 and pGE-LysRS1-DKK2-Fc were constructed from the pGE-LysRS1 vector, which is composed of the T7 promoter, LysRS gene, enterokinase recognition site, and multicloning sites (KpnI-BamHI-EcoRV-SalI-HindIII) [28]. The PCR products DKK2 and DKK2-Fc, each containing the TEV protease recognition site at the N-terminus, were cut with BamHI/SalI and cloned into the BamHI/SalI sites of the pGE-LysRS1 vector, yielding the plasmids pGE-LysRS1-DKK2 and pGE-LysRS1- DKK2-Fc, respectively. These plasmids contain the ampicillin resistance gene as a selection marker. The plasmids were cut with NdeI/HindIII, and the released DNA fragments were inserted into the NdeI/HindIII sites of the pGE-LysRS4 vector, yielding plasmids pGE-LysRS4-DKK2 and pGE-LysRS4-DKK2-Fc, respectively. The plasmid pGE-LysRS4, a modified version of pGE-LysRS1, carries the expression cassette that comprises the LysRS gene, TEV protease recognition site, multicloning sites (KpnI-BamHI-EcoRV-SalI-HindIII), and histidine tag. It also contains the kanamycin-resistance gene as a selection marker. 

The expression plasmids pGE-DKK2 and pGE-DKK2-Fc encoding DKK2 and DKK2-Fc, respectively, were constructed from the pGE-LysRS4 vector. DKK2 and DKK2-Fc PCR fragments were digested with NdeI/SalI and ligated into the pGE-LysRS4 vector cleaved with the same restriction enzymes, yielding plasmids pGE-DKK2 and pGE-DKK2-Fc, respectively. Protein expression from these plasmids is under the control of the T7 promoter, which is triggered by IPTG (Sigma-Aldrich, St. Louis, MO, USA) [28].

### 4.3. Protein Expression

Each vector with kanamycin resistance was transformed into BL21 Star™(DE3)pLysS *E. coli* (Invitrogen, Carlsbad, CA, USA). A single colony of transformants was inoculated into 3 mL LB medium containing kanamycin (7.5 µg/mL) and chloramphenicol (34 µg/mL). Cells were grown at 37 °C with vigorous shaking (180 rpm) for ~16 h. Then, 1 mL of the culture medium was diluted with 15 mL fresh LB containing antibiotics and cultured until the A_600_ reached ~0.5. After addition of 1 mM IPTG to induce recombinant protein expression, the cells were cultured at 30 °C for 4 h or 16 °C overnight. Then, 10 mL of the culture was centrifuged at 1977× *g* for 15 min, and pelleted cells were resuspended in 0.3 mL of PBS, pH 7.4, and lysed by sonication. The cell lysate was distributed into total, soluble, and insoluble pellet fractions by centrifugation at 15,000× *g* for 15 min. Each protein sample was mixed with an equal volume of 2× SDS gel-loading buffer (100 mM Tris-Cl, pH 6.8, 20% glycerol, 4% SDS, 200 mM dithiothreitol (DTT), and 0.2% bromophenol blue) and boiled at 100 °C for 2 min [28,77]. The samples were separated by SDS-PAGE and visualized by staining with Coomassie Brilliant Blue.

### 4.4. Western Blot Analysis

Western blot analysis was performed to confirm recombinant protein expression. After performing SDS-PAGE, proteins were transferred onto activated PVDF membranes using a Trans-Blot^®^ SD semi-dry transfer cell (Bio-Rad Laboratories, Hercules, CA, USA) in Towbin buffer (Bio-Rad Laboratories) for 80 min at 60 mA. The membranes were blocked with 5% skim milk in Tris-buffered saline, pH 7.4, containing 0.1% Tween 20 (TBST) overnight at 4 °C. After washing the membrane thrice with TBST, the membranes were incubated with anti-DKK2 (H-140) antibody (1:1000 in TBST; Santa Cruz Biotechnology, Dallas, TX, USA) for 2 h, followed by washing thrice and incubating with anti-rabbit-IgG Ab conjugated horseradish peroxidase (1:10,000 in TBST; Sigma-Aldrich) for 1 h. After washing thrice, the membranes were treated with WEST-ZOL (Intron, Seongnam, Korea) and exposed to X-ray film in a dark room.

For detection of phosphorylated β-catenin and non-phosphorylated (active) β-catenin, HEK 293T cells were seeded in 6-well culture plates. After 24 h, each well was treated with 500 ng LysRS, 500 ng LysRS-DKK2-Fc, 500 ng TEV-cleaved LysRS-DKK2-Fc, 10 µM IWR-1 (ApexBio, Hsinchu City, Taiwan), or PBS for 3 h before transfection with 500 ng of β-catenin and TCF4 plasmid using Lipofectamine 2000 (Invitrogen). At 24 h post-transfection, cells were treated with 500 ng of LysRS, 500 ng LysRS-DKK2-Fc, 500 ng TEV-cleaved LysRS-DKK2-Fc, 10 µM of IWR-1, or PBS. After 24 h, cells were lysed with RIPA buffer (Thermo Fisher Scientific, Waltham, MA, USA) on ice for 30 min. Cell lysates were separated by SDS-PAGE and transferred onto activated PVDF membranes. After blocking with 3% BSA in TBST for 1 h at 25 °C, the membranes were incubated with a primary antibody against phospho-β-catenin (Ser33/Ser37/Thr41) (1:1000; Cell Signaling Technology, Danvers, MA, USA), non-phospho (active) β-catenin (Ser33/Ser37/Thr41) (1:1000; Cell Signaling Technology) or β-actin (1:5000; Sigma-Aldrich) in TBST overnight at 4 °C. After washing thrice with TBST, membranes were incubated with anti-rabbit-IgG Ab conjugated horseradish peroxidase (1:10,000 in TBST; Sigma-Aldrich). After washing thrice, the membranes were treated with WEST-ZOL and imaged with X-ray film as described above [78,79].

### 4.5. Purification and Quantification of Recombinant Protein

For purification of LysRS-DKK2-Fc, Protein A affinity chromatography was performed using the ÄKTAprime system (GE Healthcare Life Sciences, Pittsburgh, PA, USA). A volume of 500 mL culture of induced transformants was centrifuged at 1977× *g* for 15 min, and the cells were lysed in 8 mL binding buffer (20 mM sodium phosphate containing 1 mM PMSF; Sigma-Aldrich) by sonication. The soluble fraction was obtained by centrifuging the lysate at 15,000× *g* for 15 min, and the supernatant was applied to an equilibrated HiTrap™ Protein A HP column (GE Healthcare Life Sciences). After sufficient washing with binding buffer, proteins were eluted by gradually decreasing the pH by mixing binding buffer with elution buffer (0.1 M citric acid, pH 3.0).

The eluted fractions were pooled and dialyzed against PBS. Purified proteins were concentrated with Amicon^®^ Centriprep filters (Millipore, Burlington, MA, USA) and quantified using a gel densitometer, with BSA used as the standard. After SDS-PAGE, the gel was stained with Coomassie blue. After destaining, the gel image was captured by an image analyzer, and the density of each band was analyzed using BIO-1D image analysis software (Vilber, Seoul, Korea). The proteins were stored at −80 °C until use.

For purification of LysRS protein, Ni-affinity chromatography was performed as previously reported [28]. The protocol was similar to that for Protein A affinity chromatography. However, we used a HiTrap™ Ni-NTA column (GE Healthcare Life Sciences), and the binding buffer consisted of 50 mM Tris-Cl, pH 7.5, 300 mM NaCl, 10% glycerol, 2 mM β-mercaptoethanol, 0.05% Tween-20, and 10 mM imidazole. The elution buffer was the same as the binding buffer, except it contained 300 mM imidazole.

### 4.6. Cleavage of Fusion Proteins with TEV Protease

Purified LysRS-DKK2-Fc proteins were treated with recombinant TEV protease (Invitrogen) in cleavage buffer (50 mM Tris-Cl, pH 8.0, 9.0, 9.5, and 10.0, 0.5 mM EDTA, and 1 mM DTT) or various excipients (50 or 100 mM NaCl, 0.5 M arginine, 1 M urea, 0.5 M trehalose, 10 mM PEG, 1 M guanidine hydrochloride, 10% glycerol, or 0.5% Tween-20) [33,34,35,36,37,38,39,40] in diverse conditions as described in the Section 2. The cleavage of LysRS-DKK2-Fc proteins and solubility of TEV-cleaved DKK2-Fc proteins were analyzed by SDS-PAGE. The quantification of DKK2-Fc proteins was performed as described above.

### 4.7. Stability Analysis of Recombinant Proteins

For the stability analysis of fusion proteins and TEV-cleaved proteins, proteins were stored for up to five days at 4 and 25 °C, respectively. Stored proteins were distributed into total, soluble, and insoluble pellet fractions by centrifugation at 13,000× *g* for 15 min. The stability of proteins was determined by SDS-PAGE analysis.

### 4.8. TOPflash Reporter Assay

For the TOPflash reporter assay, HEK 293T cells were maintained in Dulbecco’s modified Eagle’s medium (DMEM) supplemented with 10% heat-inactivated fetal bovine serum (FBS), 1% penicillin, and 1% streptomycin (Gibco, Thermo Fisher Scientific). Cells were plated at 2 × 10^4^ cells/well in 12-well plates 24 h before transfection, and each well was treated with PBS, LysRS, LysRS-DKK2-Fc, TEV-cleaved LysRS-DKK2-Fc, or IWR-1 for 3 h prior to transfection. Cells were transfected with DNA mixtures (500 ng β-catenin, TCF4, and TOPflash reporter plasmid) and 0.2 μg β-galactosidase reporter gene using Lipofectamine 2000. At 18 h post-transfection, cells were treated with PBS, LysRS, LysRS-DKK2-Fc, TEV-cleaved LysRS-DKK2-Fc (1), TEV-cleaved LysRS-DKK2-Fc (2), or IWR-1. After 6 h, cells were lysed with Promega Lysis Buffer (Promega, Madison, WI, USA), and TOP luciferase activity was measured using the Promega Luciferase Assay System [42,45]. 

### 4.9. Co-Immunoprecipitation

Proteins were subjected to immunoprecipitation with anti-LRP6 antibody (R&D Systems, Minneapolis, MN) using Protein G agarose (Roche Diagnostics, Indianapolis, IN, USA). Co-immunoprecipitated proteins were analyzed by western blotting using anti-LRP6 or anti-DKK2 antibody (Santa Cruz Biotechnology) [62,63]. DKK2 and LRP6 proteins were obtained from R&D Systems. 

### 4.10. Tube Formation Assay

The biological activity of LysRS-DKK2-Fc and TEV-cleaved LysRS-DKK2-Fc was estimated using tube formation assay in HUVECs (Bio4You, Seoul, Korea) [80]. HUVECs were suspended in M199 medium supplemented with 0.1% BSA. An aliquot of 500 μL cell suspension was added to each well of a BD Matrigel™ Matrix 12-well plate (BD Biosciences, San Jose, CA, USA). Next, each well was treated with 50 or 100 ng/mL protein (LysRS, LysRS-DKK2-Fc, or TEV-cleaved LysRS-DKK2-Fc) for 16 h in M199 medium. The cells cultured on the plate were fixed in HBSS medium containing 1% paraformaldehyde and then stained with 10 mM Calcein AM (BioVision, Milpitas, CA, USA) for 30 min. The cells were washed in PBS and observed by optical microscopy to quantify the number of branch points. 

### 4.11. In Vivo Matrigel Plug Assay

An aliquot of 600 μL liquid Matrigel at 4 °C was mixed with 14 nM LysRS-DKK2-Fc or TEV-cleaved LysRS-DKK2-Fc and injected into the abdominal subcutaneous tissues of seven-week-old male C57BL/6 mice. The injected Matrigel rapidly formed a solid gel plug. After three days, the mouse skin was pulled back to expose the Matrigel plug and quantify blood vessel infiltration. Hb levels were measured using the Drabkin Reagent Kit 525 (Sigma-Aldrich) [13]. Animal experiment was approved by the institutional animal care and use committee (IACUC) of Yonsei University (IACUC-A-201801-158-01).

## 5. Conclusions

RBPs stabilize disordered proteinaceous materials for acquiring and maintaining their structural stability and functional competence. Warranting further investigation of DKK2 as Wnt signaling antagonist, the present approach is conducive to accelerating the functional validation of ‘difficult-to-express’ proteins and enhance the druggability of the repertoire of IDPs from human proteome.

## Supplementary Materials

Supplementary materials can be found at https://www.mdpi.com/1422-0067/20/11/2847/s1.

## Abbreviations

IDP	intrinsically disordered protein
RBP	RNA-binding protein
Fc	fragment crystallizable domain of immunoglobulin
IDRs	intrinsically disordered regions
DKK2	Dickkopf2
LysRS	lysyl-tRNA synthetase
EC	endothelial cell
MBP	maltose-binding protein
TEV	Tobacco etch virus
PEG	polyethylene glycol
TCF4	T cell-specific factor 4
Fz	Frizzled
LRP	low-density lipoprotein receptor-related protein
DBPs	DNA-binding proteins
RBDs	RNA-binding domains
