# Supplementary material for: Stabilization of Intrinsically Disordered DKK2 Protein by Fusion to RNA-Binding Domain
Source: Int J Mol Sci. 2019 Jun 11;20(11):2847. doi: 10.3390/ijms20112847 (PMC6600415; doi:10.3390/ijms20112847)
Supplement: Supplementary file 1 [file ijms-20-02847-s001.pdf]

A

B

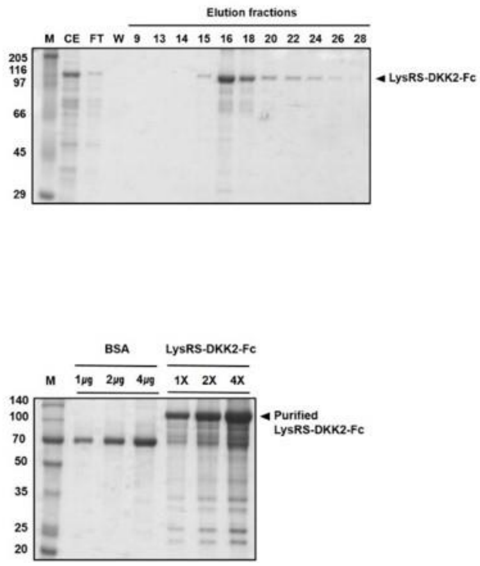

Figure S1. Purification and quantification of DKK2-Fc fusion protein. (**A**) Purification of LysRS-DKK2-Fc by affinity chromatography. CE, FT, and W represent crude extract, flow-through, and washing fraction, respectively. (**B**) Quantification of the purified LysRS-DKK2-Fc. The overall purification yield was ~ 2 mg from 1 L of *E. coli* culture.

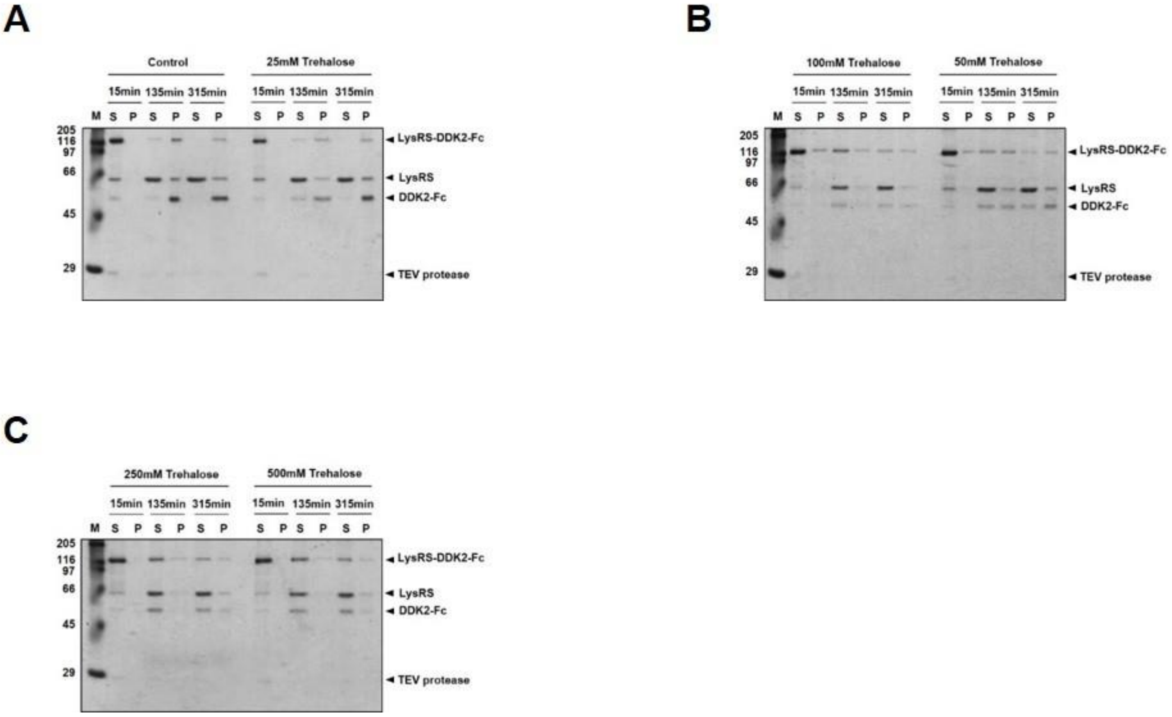

Figure S2. TEV protease cleavage of LysRS-DKK2-Fc fusion protein by treating with increasing concentrations of trehalose. (**A–C**) The fusion protein was cleaved by TEV protease with 25, 50, 100, 250, or 500 mM trehalose in pH 9.0 buffer at 25°C.

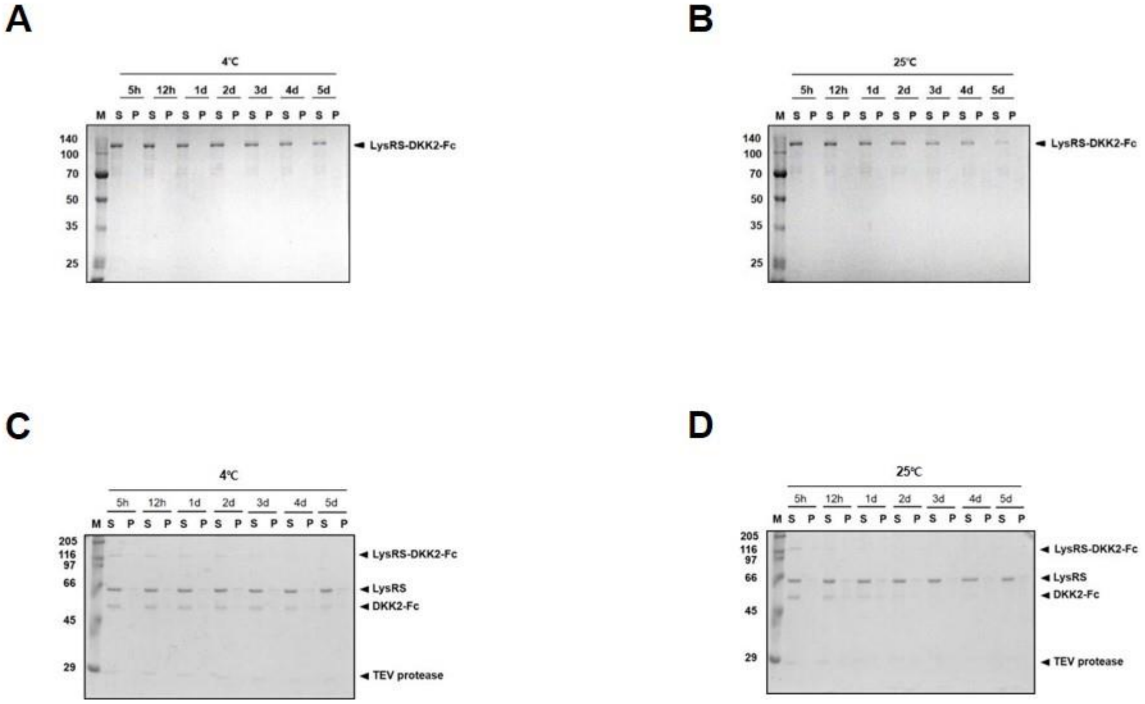

Figure S3. Stability of DKK2 in diverse storage conditions. LysRS-DKK2-Fc and TEV-cleaved LysRS-DKK2-Fc were stored for up to 5 days at 4°C (**A** and **C**, respectively) and 25°C (**B** and **D**, respectively).
